# Editorial: Nutritional status assessment and its links with chronic disease prognosis and surgical outcomes

**DOI:** 10.3389/fnut.2024.1481810

**Published:** 2024-10-23

**Authors:** Gabriela Villaça Chaves, Barbara Perez Vogt, Geórgia das Graças Pena, Rodolfo Espinoza

**Affiliations:** ^1^Division of Surveillance and Analysis, National Cancer Institute (INCA), Rio de Janeiro, Brazil; ^2^Nutrition Department, Federal University of Uberlandia, Uberlândia, Minas Gerais, Brazil; ^3^Medical Science Postgraduation, Instituto Dor, Rio de Janeiro, Brazil

**Keywords:** nutritional status, nutritional screening, prognosis, surgical complications, survival

Nutritional disorders are closely linked to poor health outcomes including prolonged hospital stays, postoperative complications, cancer treatment toxicity, shorter survival, and reduced quality of life (1). While the use of nutritional assessment tools for both diagnostic and outcome measurement purposes has been widely explored, the interplay among various nutritional disorders remains under-explored in the literature. Special attention should be given to screening markers and those that are easily applicable in clinical practice, such as the Prognostic Nutritional Index (PNI) and the GNRI (Geriatric Nutritional Risk Index), which offer practical, bedside solutions for assessing patient risk (2–4).

This Research Topic aimed to provide a comprehensive update on the scientific evidence regarding nutritional status assessment tools as prognostic indicators for clinical and surgical outcomes.

In this Research Topic, 17 papers cover the aforementioned aspects, exploring these tools in diverse clinical settings. These studies elucidate the potential of nutritional and body composition indices to improve patient outcomes through more personalized and targeted interventions.

Several studies in this Research Topic emphasize the critical role of body composition in predicting clinical outcomes, particularly in cancer patients. Sarcopenia and sarcopenic obesity were found to be significantly associated with poor overall survival and recurrence-free survival in patients with primary liver cancer (Li et al.). Moreover, sarcopenia emerged as a significant prognostic factor for shortened survival following pancreatectomy, highlighting its link to an elevated risk of mortality (Zhong et al.). These findings underscore the need for further research to clarify how sarcopenia influences long-term outcomes after cancer-related surgeries.

The importance of body composition was further reinforced by studies on colorectal cancer and abdominal surgery. The Cancer Cachexia Index and the cachexia index based on hand-grip strength (H-CXI) were identified as superior prognostic tools for predicting postoperative outcomes in colorectal cancer patients with H-CXI showing particular effectiveness for short-term clinical outcomes (Yan et al.). Additionally, muscle atrophy and high subcutaneous adipose tissue (SAT) radiodensity were associated with poor prognosis in patients with hepatocellular carcinoma, suggesting that targeted interventions such as nutritional therapy and exercise may help improve outcomes in this high-risk group (Ohara et al.).

Of particular note is the research on novel methods for assessing muscle mass, with ultrasound (US) highlighted as a feasible alternative to computed tomography, especially when enhanced by advanced measuring software (Palmas et al.). This approach could have significant implications for clinical practice by making muscle mass assessment more accessible, less invasive, and easier to perform.

The critical role of various nutritional indices as prognostic tools across different clinical settings was highlighted in our Research Topic. The prognostic significance of the pan-immune-inflamation value (PIV) was consistently demonstrated across different geographical regions, tumor stages, and treatment strategies, with sensitivity analyses confirming the stability of these results (Hai-Jing et al.). Similarly, the Geriatric Nutritional Risk Index (GNRI) emerged as a valuable predictor, not only of immunotherapy response in cancer patients (Zhang et al.) but also as an effective tool for stratifying patients undergoing hemodialysis on a global scale (Hung et al.). In parallel, the PNI was found to offer superior predictive value for adverse outcomes, particularly in patients with diabetic kidney disease, where it exhibited a stronger correlation with renal histologic changes compared to other nutritional scores (Xing et al.). Further expanding on the significance of nutritional screening, malnutrition risk was shown to significantly increase the likelihood of heart disease in middle-aged Koreans (Park and Bu). This finding underscores the need for larger studies to further validate the GNRI's efficacy in predicting disease risk in the general adult population. Additionally, a simplified nutritional prognostic score, ALTA, was developed specifically for patients with HBV-related acute-on-chronic liver failure, proving to be superior in predicting short-term mortality compared to existing scores (Song et al.).

Regarding the contributions that explored the role of dietary intake, particularly antioxidant-rich diets, in health outcomes, one study investigated the relationship between the Composite Dietary Antioxidant Index (CDAI) and mortality among adults with hypertension. The findings revealed that a higher CDAI was associated with reduced all-cause, cardiovascular, and cancer mortality, underscoring the potential benefits of an antioxidant-rich diet in improving outcomes for hypertensive individuals (Zhou et al.). Another study focused on the relationship between CDAI and hyperlipidemia, utilizing data from the National Health and Nutrition Examination Survey (NHANES; Qin et al.). The results indicated a linear negative association between CDAI and the risk of developing hyperlipidemia, suggesting that increasing the intake of antioxidant-rich foods could be an effective strategy for preventing hyperlipidemia.

Overall, the findings in this Research Topic highlight the critical need for standardized and comprehensive nutritional assessment tools in clinical practice. By integrating these tools into patient care, healthcare providers can better predict outcomes and tailor interventions to improve long-term health across various diseases and surgical scenarios.
